# Fecal DNA analysis coupled with the sighting records re-expanded a known distribution of dugongs in Ryukyu Islands after half a century

**DOI:** 10.1038/s41598-024-58674-8

**Published:** 2024-04-04

**Authors:** Hiroyuki Ozawa, Takahiro Yoshihama, Shogo Gishitomi, Natsuki Watanabe, Kotaro Ichikawa, Keiichi Sato, Kenta Watanabe, Katsuhiko Takano, Yosuke Ochiai, Hiroki Yamanaka, Atsushi Maruyama

**Affiliations:** 1 Incorporated Foundation Okinawa Prefecture Environment Science Center, Urasoe, Okinawa 901-2111 Japan; 2 Kanizou Co. Ltd. Miyakojima, Okinawa, 906-0000 Japan; 3 Field Science Education and Research Center, Kyoto University, Kyoto, Kyoto 606-8502 Japan; 4 Okinawa Churashima Foundation, Kunigami, Okinawa 905-0206 Japan; 5 National Institute of Technology, Okinawa College, Nago, Okinawa 905-2192 Japan; 6 Japan Broadcasting Corporation, Shibuya, Tokyo 150-8001 Japan; 7 Center for Biodiversity Science, Ryukoku University, Otsu, Shiga 520-2194 Japan; 8 Faculty of Advanced Science and Technology, Ryukoku University, Otsu, Shiga 520-2194 Japan

**Keywords:** Biogeography, Conservation biology, Ecology

## Abstract

DNA analysis of large herbivore feces samples collected from seagrass beds at two distant sites (Irabu Island in Miyako Islands and Kushi in Okinawa Island) in the Ryukyu Islands proved that some of these feces were from dugongs, which had been treated in recent studies as extinct in this region since the last stranding of a deceased individual in 2019. In addition, local knowledge of sightings of animals thought to be dugongs and confirmed cases of dugong feeding trails since 2010 were compiled to estimate its recent distribution. This is the first scientific report on the presence of this mammal in the Ryukyu Islands within the last four years, and particularly in the Miyako Islands within the last half-century. As the Ryukyu Islands are known to be the northern limit of the dugong’s fragmented distribution in East Asia, conservation efforts are therefore needed.

## Introduction

Extinction is the irreversible termination of a taxon or population, and should be determined when there is no reasonable doubt that the last individual of the focal taxon or population has died^1^. The International Union for the Conservation of Nature (IUCN) stated that a taxon is presumed extinct when exhaustive surveys in known and/or expected habitat throughout its historic range, at appropriate times (diurnal, seasonal, annual) over a time frame appropriate to the taxon’s life cycle and life form have failed to record an individual. Errors in determining extinction lead to weakening and delaying conservation measures of the focal organism. Especially in the case of umbrella or flagship species whose requirements include those of many other species^2^, the impact of the errors is expected to extend to other species.

**Figure 1:**
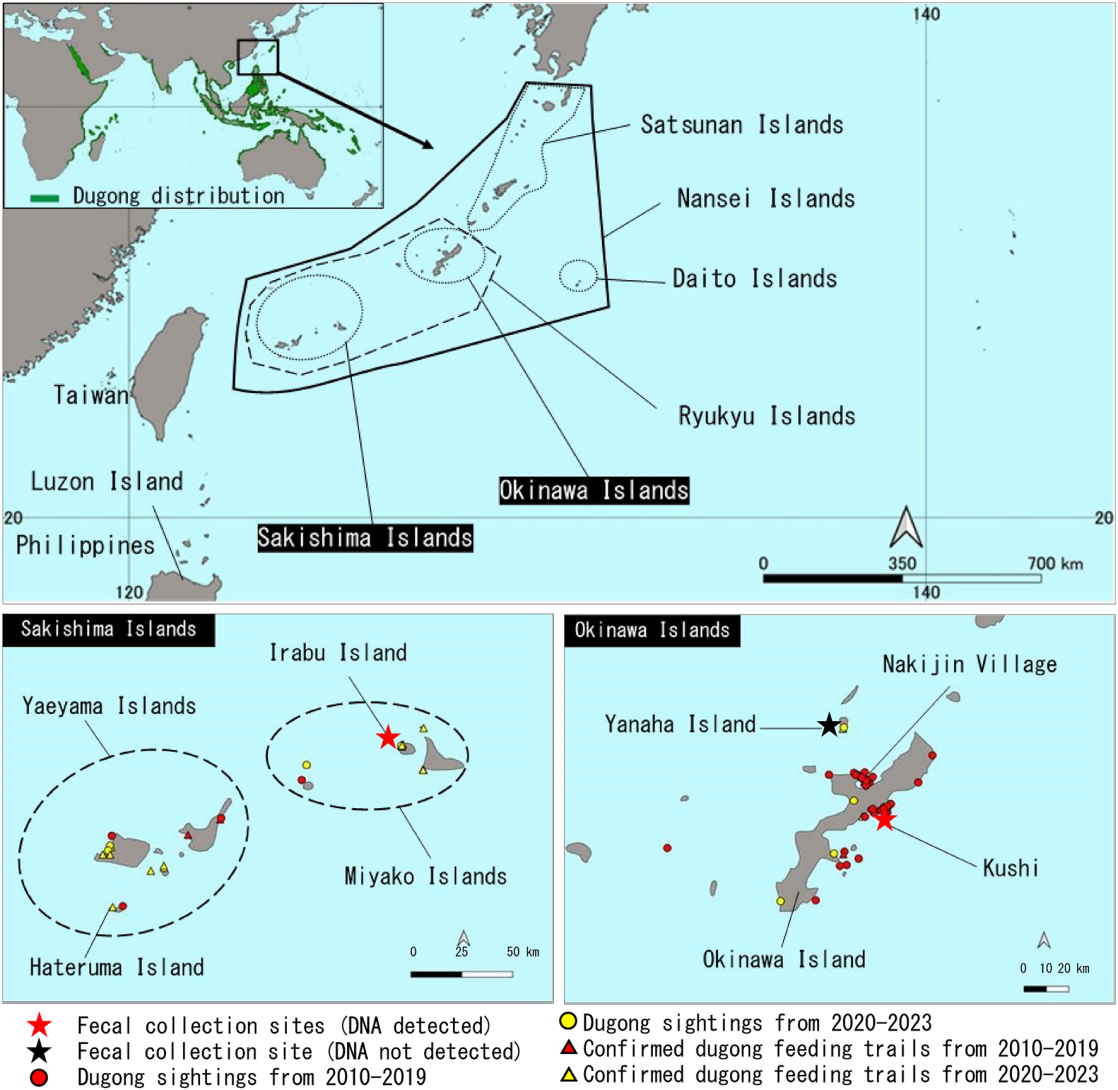
Locations where feces of large herbivores were collected, animals thought to be dugongs were sighted, and dugong feeding trails were confirmed. The worldwide dugong distribution map at the top left is based on Marsh & Sobtzick, 2019^1^.

The dugong (*Dugong dugon*), a marine herbivorous mammal of medium size, is such an umbrella species, exclusively feeding on seagrasses and hence requiring large seagrass zones in the warm shallow waters. This is the only living species of the once-diverse family Dugongidae that is listed as vulnerable to extinction on IUCN Red List^3^. This species is distributed across the Indo-West Pacific, but its current distribution is fragmented and threatened. Particularly around the northern limit of its range in East Asia (namely the Nansei Islands; Fig. 1), the subpopulation size has continuously declined from the eighteenth century to the early twentieth century mainly due to overhunting^4^; thus, IUCN and local government have classified it as Critically Endangered and Endangered, respectively^5,6^. Furthermore, there have been no direct observations of dugongs in the Ryukyu Islands since a dead adult female was discovered off the coast of Nakijin Village, on the northwestern part of Okinawa Island in 2019.

**Figure 2:**
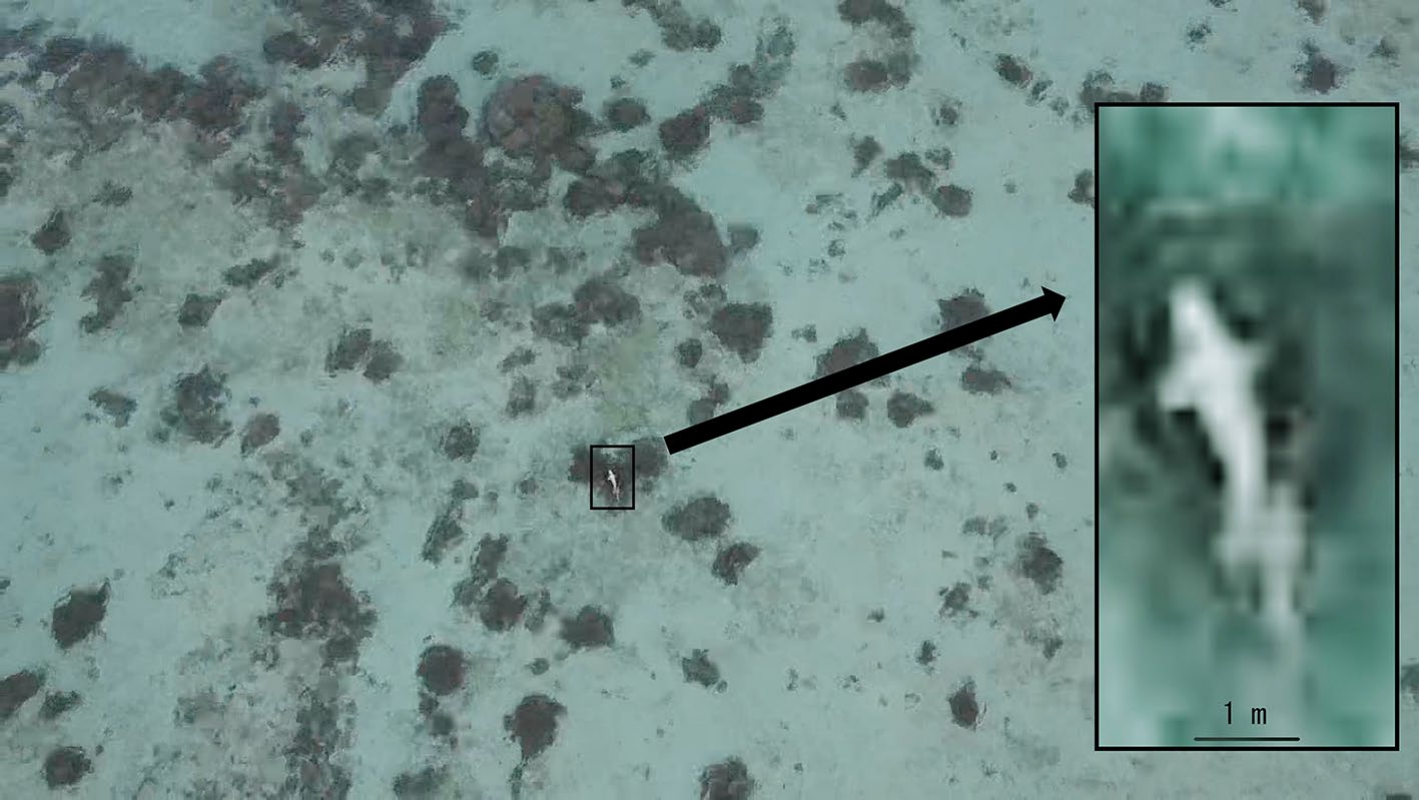
A photograph of a marine animal thought to be a dugong in a coastal area of Irabu Island on 26 September 2019, taken with UAV by Takahiro Yoshihama, the second author of this paper.

A recent study of population dynamics, based on fishery statistics, local newspaper articles, and aerial observations, stated that this mammal possibly became extinct in 2019^7^. This study has been cited by at least one academic paper that treated the dugong within the region extinct^8^, contrary to the IUCN statement^1^. Such quick and poor determination of extinction would allow further destruction of its habitat. In fact, local coastal fishermen and residents have reported sightings of animals thought to be dugongs^9^ (Fig. 2). Furthermore, local researchers, including the first author of this paper, have conducted a number of field surveys for dugong feeding trails (large unvegetated tracks left by dugongs after feeding in seagrass beds^10^). Such local knowledge has potential to provide worthy evidence to understand its current distribution, especially when combined with scientific evidence such as DNA fragment detection.

In this study, we analyzed feces of large herbivores collected from seagrass beds in the Ryukyu Islands, using a species-specific PCR assay designed for dugongs. In addition, we rigorously examined records from 2010 to 2023 of confirmed dugong feeding trails as well as dugong sightings. The collected information is useful to reconsider the current distribution of this endangered mammal.

## Results

### Analysis of fecal DNA

**Figure 3:**
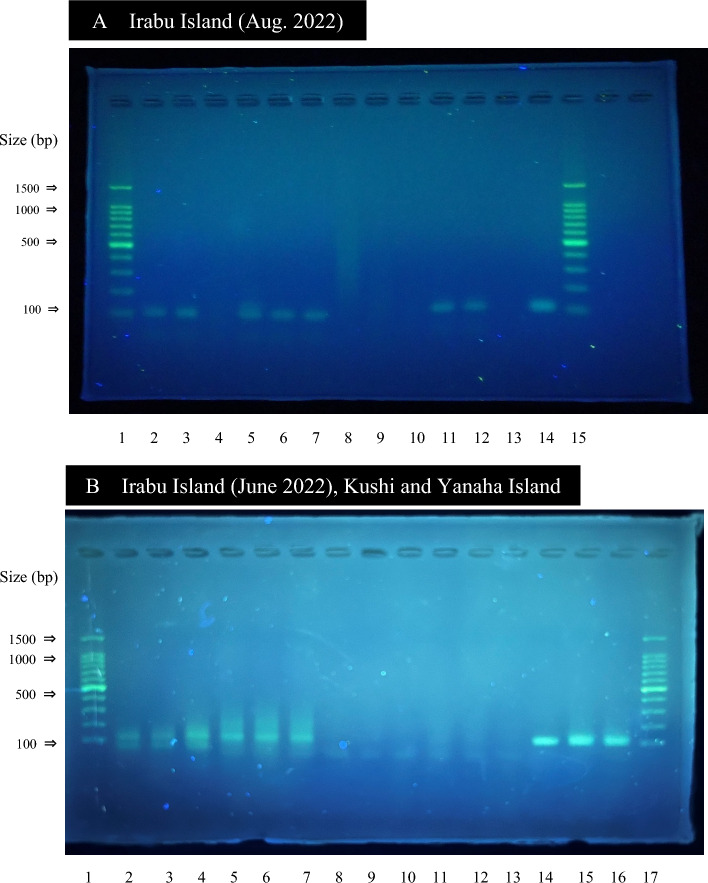
Photographs of agarose gel electrophoresis patterns of amplicons after PCR of marine mammal’s feces using dugong-specific primers. Lanes A1 and A15: DNA ladders, A2-12: Irabu collected in August of 2022, A13: negative controls, A14: positive controls, B1 and B17: DNA ladders, B2-4: Irabu collected in June of 2022, B5-7: Kushi, B8-10: Yanaha, B11-13: negative controls, B14-16: positive controls. Each lane stands for each replicate of extracted DNA from feces samples. Single and three replications were made in the DNA extraction for each feces sample in (**A**) and (**B**), respectively.

Among the feces collected from the seagrass beds on the western shore of Irabu Island (hereafter, Irabu), the Kushi shore of Okinawa Island (Kushi), and the Yanaha shore of Yanaha Island (Yanaha), fragments of the D-loop region of the mitochondrial DNA unique to dugongs were detected from the Irabu and Kushi samples by PCR assays using a species-specific primer set, followed by the electrophoresis (Table 1; Fig. 3). These results indicated that these feces were from dugongs.

**Table 1:** Results of dugong-specific PCR assay and Sanger sequence analysis of DNA extracted from marine mammal's feces.

Site	Collection date	PCR positive	Sequence positive	Sequence identity
Irabu Island	13 Jun 2022	3/3 (100%)	3/3 (100%)	≥ 100%
Kushi, Okinawa Island	7 Jul 2022	3/3 (100%)	3/3 (100%)	≥ 100%
Yanaha Island	17 Jul 2022	0/3 (0%)	N/A	N/A
Irabu Island	23 Aug 2022	7/11 (64%)	7/7 (100%)	≥ 100%

N/A: DNA sequence analysis was not conducted.

Specifically, from Irabu, we collected one fecal sample from a seagrass bed in June 2022. PCR tests on the three replicates of extracted DNA from the feces sample were all positive using the dugong-specific primer set. Also from Irabu, we obtained 11 feces samples as bulks of two to five feces concurrently found at each of 11 different locations of seagrass beds in August 2022. DNA was extracted from the samples with single replication and subjected to PCR tests. At that time, seven out of the 11 samples were positive. From Kushi, we collected one sample that pooled two feces found concurrently in July 2022, and PCR results for triplicated extracted DNA from the pooled sample were all positive. From Yanaha, we collected one fecal sample in July 2022, and all PCR results were negative for triplicates of the extracted DNA.

In all PCR analyses, dugong-specific DNA was successfully amplified in the positive controls (DNA extracted from the muscle tissue of a dugong carcass), but not in the negative controls (ultrapure water). The PCR amplicons of Irabu and Kushi samples that yielded positive results were Sanger-sequenced, and BLAST searches confirmed that the sequences of all these samples were 100% identical to dugong sequences (e.g., BLAST: MK986817 in India, KJ022758 in Thailand, MT136733 and KJ944386 in Australia).

### Sighting records of animals thought to be dugongs and confirmed cases of feeding trails of dugongs

**Figure 4:**
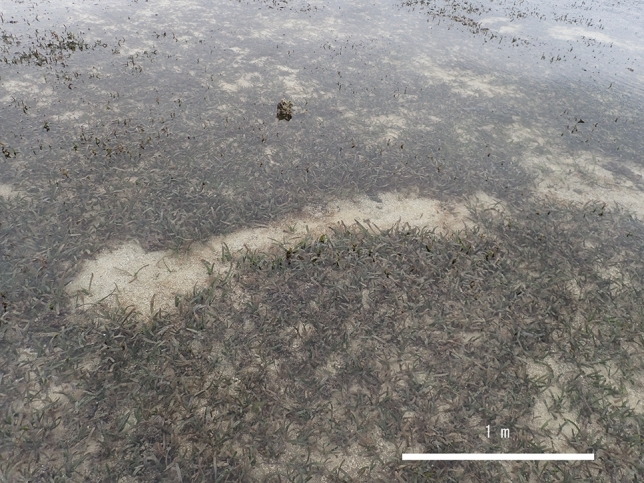
A photograph of a dugong feeding trail confirmed in a coastal area of Irabu Island in March 2020.

Our examination of the local knowledge since 2010 (Supplementary Table S1) found 66 sightings of animals thought to be dugongs and 26 confirmed cases of feeding trails in a wide range of coastal areas of the Ryukyu Islands between 2010 and 2023 (Fig. 1). Feeding trails are linear feeding trails of < 30 cm width that dugongs leave in the seagrass beds after feeding on seagrass (Fig. 4)^11^, and have been used for studying dugong distribution especially in the regions where this mammal is rare^9,12^. Sightings were intensively recorded around Okinawa Island before 2019, while the number of feeding trails found in the Sakishima Islands increased after 2020. Among the sighting records, multiple large marine animals thought to be dugongs were sighted near Hateruma Island in 2018 and near Irabu Island in 2019. In the Hateruma case, one small individual seemed to be pulling its head to the side of a large individual was sighted from a helicopter (described in a government document^13^).

## Discussion

The IUCN-listed Japanese dugong subpopulation in the Nansei Islands had been estimated to have less than 10 mature individuals^5^. There have been no confirmed cases of dugong inhabitation since 2019, when the dead adult female was found in Nakijin Village on the northwestern of Okinawa Island (Fig. 1). The dugong subpopulation around Okinawa Island was even estimated to be near extinct or extinct^5^. Citing this study, Lin et al.^8^ even stated that the Japanese dugong became extinct in 2019. However, it was obviously too quick and poor to conclude its extinction, because there have been continuous sightings of animals thought to be dugongs by local coastal fishermen and others in this region since 2010, and even after 2020, as our study has shown. Dugong feeding trails have been also confirmed (Fig. 1). Furthermore, our detection of dugong DNA using a dugong-specific PCR assay in the feces of large herbivores collected at the two distant sites, namely Irabu in the Miyako Islands and Kushi in Okinawa Island, strongly supports the possibility that dugongs still inhabit a wide area in the Ryukyu Islands, and that one should never conclude that dugongs are extinct within the region.

Our dugong DNA detection in Irabu samples is the first confirmation of dugong inhabitation in the Miyako Islands in approximately half a century after the captures in the late 1960s (1967 and 1968)^6^. We have no data to judge if the dugong has been continuously distributed or it recently recovered in Miyako Islands. For several decades, the distribution of the dugong in the Ryukyu Islands was thought to be limited to a small area around Okinawa Island^14^. Importantly, however, we detected dugong DNA in fecal samples collected from Irabu Island twice within two months (June and August of 2022). This coupled with continued observations of feeding trails and reports of dugong sightings around Irabu Island since 2019^9^ suggests that dugongs are not short-term visitors but have settled in the surrounding waters. The sighting records included observation of an adult and a small individual, which is thought to be a mother and calf, in Hateruma Island, located at the southwestern tip of the Sakishima Islands. This observation strongly suggests that the Sakishima Islands are important for the dugongs’ reproduction.

Our dugong DNA detection should highly affect the local conservation policy. With the evidence of dugongs’ presence, governments and other relevant agencies should never weaken or delay conservation measures of this umbrella species of seagrass zones in the warm shallow waters. Optimal dugong conservation tactics depend on the size of its home range. If this mammal has settled for a considerable period of time in limited areas, protection measures such as preventing bycatch by fishing nets around those areas should be implemented as soon as possible. If this mammal migrates long distance frequently, seagrass beds should be widely protected to ease their migration. At this point, our knowledge of the migratory ecology of this subpopulation in the Nansei Islands is very limited, which accounts for the difficulty in estimating its population size. Although recent studies on the long-distance migration of dugongs have been discussed in detail by Deutsch et al.^15^, easy extrapolation should be refrained from and conservation policies should be adopted that take both possibilities (settlement and migration) into account.

The dugong subpopulation in the Ryukyu Islands is endangered due to the rapid and almost irreversible decline in population size in the early twentieth century resulting from overhunting with dynamite^3^. However, our study suggests that more than 100 years after this rapid decline, dugongs are still likely to be present across a wide area of the Ryukyu Islands. This suggests the possibility that a certain number of dugongs may have continuously inhabited the coastal areas of these islands and surprisingly maintained the population for approximately a century even after the decline due to the impact of overhunting.

How then has the dugong population been maintained in the Ryukyu Islands? One hypothesis is that the Philippines, which has a healthier dugong population than the Ryukyu Islands, has served as the source of the dugongs, meaning that dugongs migrate from the Philippines to the Ryukyu Islands on rare occasions. Dugongs have the ability to migrate long distances in a short period of time, with a record of 140 km in two days^16^ and an average distance of 244 km in an average of 180 hours^15,17^. Although the current distribution is unknown, dugongs historically inhabited nearly every island in the Philippines^18^. The linear distance is about 400 km from the islands located north of Luzon Island in the Philippines to Hateruma Island (the southern tip of Nansei Islands), where marine animals thought to be dugongs (mother and calf) were observed in 2018 located at the southern tip of the Ryukyu Islands. In addition, there is a strong current, the Kuroshio Current, from the eastern part of Luzon Island to the southern part of the Ryukyu Islands. The velocity of the Kuroshio Current is known to reach a maximum of 7.4 km/h (178 km/day) near its axis^19^, and if dugongs migrate from northern Luzon Island to the Ryukyu Islands, it is estimated that they would reach the Ryukyu Islands within three days. In addition, mtDNA-based population genetic studies of dugongs using samples from Australia to Southeast Asia, including the Ryukyu Islands, have suggested the possibility that individuals inhabiting Okinawa and the Philippines may migrate between the two areas^20^. From the above, it is possible that such rare migration of individuals from the Philippines exists as one of the reasons for the long-term maintenance of the dugong subpopulation in the Ryukyu Islands. In the future, there is an urgent need to elucidate the migratory ecology and population structure of dugongs in the Ryukyu Islands, and to take measures to conserve the dugongs and seagrass beds that currently inhabiting the islands. Specific conservation measures include promoting education and dissemination to coastal fishermen and local residents regarding bycatch prevention and seagrass bed conservation.

## Materials and methods

### Sampling

Feces of large herbivores that may be of dugongs, were collected from seagrass beds in Irabu, Kushi, and Yanaha in the Ryukyu Islands (Fig. 1). Sampling methods were slightly different between sampling sites and dates, because feces were always found by chance.

In the Irabu seagrass beds, feces were found floating on the sea surface. One fecal sample was collected on 13 June 2022. The Irabu sample collected in June was stored in an unused zipper-top plastic bag with 100 ml of surrounding water, and 0.5 ml of 10% benzalkonium chloride solution per liter was added at the site to inhibit DNA degradation by microorganisms^21^, then stored frozen at − 20 °C until analysis. Two months later on 23 August 2022, samples were collected at 11 separate locations in seagrass beds. At each location, 2–5 feces were pooled in one sample making 11 samples in total for a total of 30 feces. Fecal samples from the different locations were stored separately in unused zippered plastic bags along with approximately 200 ml of surrounding seawater and 0.5 ml of 10% benzalkonium chloride solution. The feces samples were transferred to unused sample bottles prewashed with sodium hypochlorite solution in the laboratory, and stored at − 20 °C until analysis. The two sites where feces were collected on Irabu were seagrass beds, and several feeding trails were observed on the same day in the seagrass beds surrounding the feces collection sites.

Two feces samples were collected from the seagrass beds in Kushi, Okinawa Island on July 7, 2022. The feces were drifting several meters apart on the sea surface above the seagrass beds and stored in plastic containers on land. At this time, the fecal samples were immersed in a small amount of seawater. Then 1 ml of 10% benzalkonium chloride solution was added to the feces in the field and stored in an unused sample bottle that had previously been washed with sodium hypochlorite solution in the laboratory. The Kushi samples were stored frozen at − 20 °C until analysis. The area around the feces collection point in Kushi is a seagrass bed, but no observation of the seagrass bed was made at the time of collection, and the presence of feeding trails is not clear.

At the seagrass beds in Yanaha, one fecal sample was collected on July 17, 2022, while it was submerged on the seagrass bed floor. The Yanaha fecal sample was stored in a PET container with surrounding seawater after collection, and the solid part of the feces material was cut off in the laboratory one day after collection and stored frozen at − 20 °C until analysis. Fecal samples collected at Yanaha were collected in a seagrass bed and several feeding trails were observed in the vicinity.

### Analysis of fecal DNA

For samples collected from Irabu Island seagrass beds in August, DNA extraction was performed once per sample. For other samples, DNA extractions were replicated three times per sample. DNA was extracted from feces using the QIAamp Fast DNA Stool Mini Kit (Qiagen), following the manufacturer operating instructions. Approximately 100 mg of the surface of the solid portion of each fecal sample was collected with a sterile spatula to obtain 50 µL of the final eluate. The species-specific primers for dugong (Tol et al.^22^) were used. Primer sequences for dugong species identification are F: 5'—CGCGCGCTATGAACTTCGT—3'; R: 5'—GGGGGTAAGTAGTGTAATGCACG—3' and the product size is 110 bp.

For the analysis methods after DNA extraction, we referred to a previous study on the molecular identification of dugong and green turtle feces^22^. Extracted DNA samples were subjected to nested PCR in one well per sample. The composition of the first PCR solution was 12.5 µL of iProof HF Master Mix (Bio-Rad), 0.5 µL each of 10 µM primers, 1 µL of extracted DNA solution, and 10.5 µL of sterile water, for a total volume of 25 µL.

To increase the detection rate of DNA extracted from feces, two-step PCR were performed in which the first PCR products were diluted as second PCR templates. The primers used in the second PCR are the same as those used in the first PCR. A Biometra TOne (Analytik Jena, Germany) was used for PCR amplification. PCR was performed under the following conditions as described by Tol et al.^22^: holding at 98 °C for 45 s, and then the cycle consisting of 10 s at 98 °C, 30 s at 65 °C, and 15 s at 72 °C, was repeated 35 times, followed by an elongation reaction at 72 °C for 5 min. Ultrapure water was used as a negative control, and DNA extracted from the muscle of the dugong carcass found near the coast of Nakijin Village, Okinawa Island, in March 2019 was used as a positive control. Both negative and positive control samples were included in each PCR run. For the composition of the solution of the second PCR, 1 µL of the first PCR product was diluted 100-fold in sterile water and used as a template. The second PCR was performed under the same conditions as for the first PCR.

The products of the second PCR were visualized by electrophoresis in 2% agarose gels. A 100 bp DNA Ladder (TAKARA BIO) was used as a DNA molecular weight marker. The PCR products were cut from agarose gels, and the DNA was purified using NucleoSpin Gel and PCR Clean-up (MACHEREY–NAGEL).

The DNA was fluorescently labeled using a SupreDye v3.1 Cycle Sequencing Kit (AdvancedSeq). Purification after cycle sequencing was performed using a SupreDye XT Purification Kit (AdvancedSeq). Sanger sequence analysis was performed using a DS3000 Compact CE Sequencer (Hitachi High-Tech), and all sequences were compared to the sequence from dugongs by using BLAST (National Center for Biotechnology Information).

### Sighting records of animals thought to be dugongs and confirmed cases of feeding trails

The Japanese Ministry of the Environment conducted began surveying dugong sightings throughout Okinawa Prefecture in 2003^23^, and those surveys have continued to the present^9^. Records for this effort include cases of dugong sightings by fishermen and marine recreationists in coastal areas of the Ryukyu Islands. In addition, the Ministry of the Environment, Okinawa Prefecture, and other administrative agencies have conducted continuous diving surveys on the distribution of dugong feeding trails in seagrass beds to identify dugong habitats^9,12^. In these surveys, dugong feeding trails were defined as linear bare areas of 30 cm width or areas with seagrass grazed down to the subterranean stems^10,11^. Our study summarized the records of sightings of animals thought to be dugongs and the confirmed dugong feeding trails in the Ryukyu Islands since 2010. Note that the first author of this paper has been involved in a scientifically responsible capacity in all of the surveys mentioned in this section.

### Supplementary Information

Supplementary Tables.

## Data Availability

DDBJ accession numbers of the DNA sequences analyzed in the present study are LC780086-LC780098.
